# Application and evaluation of digital PCR platforms for same-day detection of *Vibrio parahaemolyticus* in mussel samples

**DOI:** 10.1016/j.crfs.2026.101484

**Published:** 2026-06-23

**Authors:** Ana Costa-Ribeiro, David Rocha-Grandal, Lara Pierantoni, Carlos Honrado, Lorena Diéguez, Alexandre Lamas, Alejandro Garrido-Maestu

**Affiliations:** a Laboratory of Microbiology and Technology of Marine Products (MicroTEC), Institute of Marine Research (IIM), CSIC, Eduardo Cabello, 6, Vigo, 36208, Spain; b Department of Biochemistry, Genetics and Immunology, University of Vigo, Vigo, 36310, Spain; c Department of Functional Biology and Health Sciences, University of Vigo, Vigo, 36310, Spain; d Medical Devices Research Group, International Iberian Nanotechnology Laboratory, Avenida Mestre José Veiga s/n, Braga, 4715-330, Portugal; e Food Hygiene, Inspection and Control Laboratory (LHICA), Department of Analytical Chemistry, Nutrition and Bromatology, Veterinary School, Campus Terra, Universidade de Santiago de Compostela, Lugo, 27002, Spain

**Keywords:** Digital PCR, dPCR, ddPCR, qPCR, Real-time LAMP, Colorimetric LAMP, *Vibrio parahaemolyticus*, Early detection, Seafood

## Abstract

*V. parahaemolyticus* infection is a major cause of disease associated with the consumption of raw or undercooked seafood. Given the short shelf life of this type of foods, rapid detection methods are of high interest. To reach this goal, this study compared three different molecular techniques for detecting *V. parahaemolyticus* from the primary enrichment described in the ISO standard 21872-1:2017. To do so, three different DNA amplification techniques were tested: digital PCR (three different platforms), real-time PCR, and LAMP, with its real-time and colorimetric version. The analysis of spiked mussel samples, following the specific workflows considering target multiplexing capacities of the different platforms, returned an LOD50 of 15.0 CFU/25 g after the primary enrichment for qPCR; however, they were all considered negative by digital PCR even though a few positive partitions were observed. This value was improved after the secondary enrichment, allowing to reach 3.3 CFU/25 g for qPCR and LAMP strategies. The analysis of natural samples returned minor differences among the different molecular methods. In parallel to the previous study, different chromogenic agars were compared, with Chromagar Vibrio the best one, with the highest specificity, whereas HardyCHROM had the lowest, making it unsuitable when high concentrations of interfering bacteria are expected. This study highlights the importance of appropriate sample processing/preparation for sensitive and successful performance of molecular methods, as, by itself, the sole sensitivity associated with any given detection technology is not enough for pathogen detection in food matrices.

## Highlights

• After 5 h enrichment dPCR/ddPCR did not provide consistent LOD50 results.
• Real-time and colorimetric LAMP were less sensitive after 5 h enrichment.
• The LOD50 was significantly reduced after secondary enrichment.
• CHROMagar™ Vibrio outperformed other chromogenic media.
• Sample preparation is key for successful molecular detection.

## Graphical abstract

**Figure:**
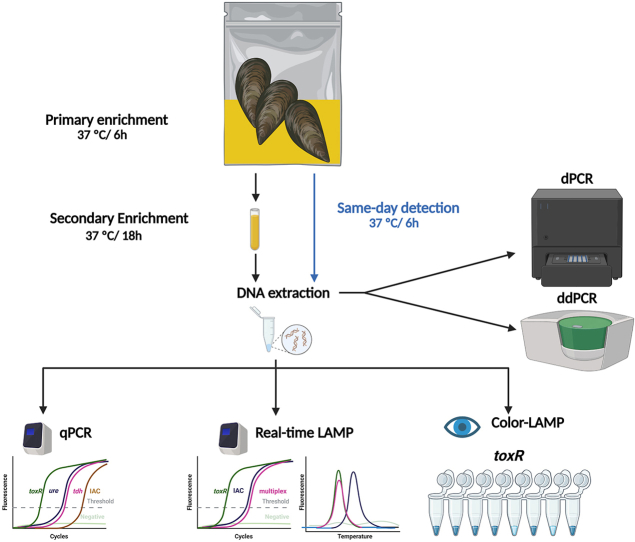

## 1 Introduction

*Vibrio* spp. are natural inhabitants of aquatic ecosystems (Baker-Austin et al., 2018). Within the genus, there are several important species, as they are well-known human and aquaculture pathogens, as well as some of increasing biotechnological interest (Farmer et al., 2015). Among the human pathogenic species, three stand out, namely *V. cholerae*, *V. vulnificus*, and *V. parahaemolyticus* (Namadi and Deng, 2023).

*V. parahaemolyticus* is the leading cause of bacterial infections associated with the consumption of raw or undercooked seafood (Baker-Austin et al., 2018; Brumfield et al., 2023; Ndraha et al., 2022). Its presence has been reported worldwide, and even a pandemic clone has been described (Martinez-Urtaza et al., 2005; Matsumoto et al., 2000; Okuda et al., 1997; D. Wang et al., 2022). The incidence of this microbe has been related to different environmental factors, such as seawater temperature and salinity, generating a seasonal pattern (D. Wang et al., 2022).

The CDC estimates that 80,000 cases of vibriosis occur annually in the United States, with 52,000 cases resulting from the consumption of contaminated food (CDC, 2024b). Previous reports have indicated that 4500 cases of *V. parahaemolyticus* infection occur annually in the United States (Wright et al., 2009). The COVIS report, which gathered data from 2019, reported 2685 vibriosis cases, of which 670 were due to *V. parahaemolyticus* (CDC, 2024a). This pathogen is regulated in countries such as the USA and China, but not in Europe (Canada, 2020; FAO, 2003; Hara-Kudo and Kumagai, 2014). ISO 21872 and Chapter 9 of the Bacteriological Analytical Manual are both official methods for *V. parahaemolyticus* detection (Charles A. Kaysner and Angelo DePaola, 2004; Hara-Kudo and Kumagai, 2014; ISO, 2017). Both methods are culture-based and, as such, they require several days of extensive culture and isolation in order to reach a final result. Moreover, when it comes to bacterial isolation, typically performed on TCBS agar and a secondary medium, the interference of other microbes, due to co-enrichment in the sample, has been reported (Ariyawansa et al., 2008; Blanco-Abad et al., 2009; Rocha-Grandal et al., 2025). In the current context of climate change and rising sea water temperatures, the concentration of *V. parahaemolyticus* in seafood may increase exponentially. Therefore, rapid methods for controlling this pathogen are necessary to maintain food safety and not compromise the marketing of these products.

DNA-based methods, more specifically Polymerase Chain Reaction (PCR) and real-time PCR (qPCR), have now been extensively applied to speed up turnaround times, given the short shelf life of certain seafoods (Gao et al., 2021; Garrido-Maestu et al., 2018; Shan et al., 2012). Recently, isothermal nucleic acid amplification has also been implemented as an additional option, with loop-mediated isothermal amplification being the most extensively applied technique (Yamazaki et al., 2010, 2011). Likewise, in recent years, digital and digital droplet PCR (dPCR/ddPCR) have emerged as more advanced alternatives to qPCR. These techniques have been reported to have higher sensitivity, among other advantages, and can detect a single copy of DNA (Kopylova et al., 2023; Salipante and Jerome, 2020).

The main goal of the present study was to determine the suitability of different dPCR/ddPCR platforms for the early detection of *V. parahaemolyticus* in seafood samples and to compare their performance with of that qPCR and LAMP. In addition to this, the suitability of novel chromogenic media for the specific isolation of *V. parahaemolyticus* was also assessed.

## 2 Materials and methods

### 2.1 Bacterial strains

*V. parahaemolyticus* strain WDCM 00037, purchased from the Spanish Type Culture Collection, was selected as the reference strain for spiking experiments. Fresh cultures were prepared by inoculating one single colony into 4 mL of Alkaline Saline Peptone Water (ASPW, Biokar Diagnostics S.A., France), and incubating it overnight at 37 °C. Final bacterial concentration was determined by measuring OD600 in a Jenway® 7205 spectrophotometer (Jenway, Staffordshire, UK), and conversion to CFU/mL made with the online application https://www.agilent.com/store/biocalculators/calcODBacterial.jsp.

### 2.2 DNA extraction

The DNA extraction procedure consisted of a thermal lysis at high temperature, along with Chelex purification. The selection of this protocol was based on its simplicity, rapid turnaround time, low cost, and previous application to the matrix under study (Rocha-Grandal et al., 2025). Briefly, a 1 mL aliquot from the ASPW primary enriched samples was centrifuged at 17000 ×g for 2 min, the supernatant was discarded, and the pellet was resuspended in 200 μL of Chelex™ 100 6 % (w/v) (Bio-Rad Laboratories, Inc., Hercules, CA, USA). To pellet potential large food particles, this step was preceded by a 1 min centrifugation at 1000 × g. The Chelex suspension was incubated at 56 °C and 1000 rpm for 15 min to avoid resin sedimentation, and subsequent bacterial lysis was performed at 100 °C and 1000 rpm for 8 min. Both incubations were performed in a TS 100 Thermo Shaker (Biosan, Riga, Latvia). Finally, the samples were centrifuged at 17000 ×g for 2 min at 4 °C, and the supernatant was transferred to a clean tube. This procedure was previously described by Abalo et al. (2024). For resource optimization, all DNA extracts were stored at −20 °C and, once defrosted for analysis, they were kept at 4 °C until needed.

### 2.3 Molecular detection

Three different DNA amplification techniques were applied in the present study, namely dPCR/ddPCR, qPCR, and LAMP. For the LAMP assay, two approaches were followed: real-time fluorescence and endpoint colorimetric detection. All primers and probes used in this work are provided in Table 1, and were purchased from Integrated DNA Technologies Inc. (IDT, Leuven, Belgium). Regardless the detection technology applied, all experiments included a Non-Template Control (NTC, nuclease-free water), a Positive Control (PC, pure *V. parahaemolyticus* DNA obtained by chelex extraction, and diluted 1/1000), and a non-competitive Internal Amplification Control (IAC, chimeric sequence artificially designed).

#### 2.3.1 dPCR/ddPCR

Three different dPCR platforms were included in the present study: Absolute Q from Thermo Fisher (AbsQ), QX600 AutoDG, and QX Continuum from BioRad, hereafter referred to as QX600 and QXC, respectively. These machines perform the partitioning of the reactions in two different ways: the AbsQ machine uses a special plate with microwells, while both ddPCR platforms generate droplets. The preparation of the reactions for the different machines was performed following the instructions of the corresponding manufacturer, no additional assay optimization was performed. Regardless of the platform selected, positive samples were considered those with 5 times more partitions positive than the negative control, considering that unexpected positive partitions may show up as previously reported (Rutsaert et al., 2018).

##### 2.3.1.1 AbsQ

The dPCR assay consisted of a duplex assay targeting the *toxR* gene of *V. parahaemolyticus* and an Internal Amplification control. The samples were analyzed in an Applied Biosystems™ QuantStudio™ Absolute Q Digital PCR System (Thermo Fisher Scientific Inc., Waltham, MA, USA). The primer concentrations were 900 and 200 nM for *toxR* and IAC, respectively, while the probe concentrations were 250 nM for *toxR* and 200 nM for the IAC, and 1000 copies of IAC DNA. In addition to this, each reaction had a final volume of 10 μL, composed of 2 μL of Absolute Q™ DNA Digital PCR Master Mix (5X) (Applied Biosystems™, Foster City, CA, USA), 3 μL of template DNA, and the remaining volume was filled with nuclease-free water (Tiaris Biosciences, Cordoba, Spain). From the 10 μL reaction mixture, 9 μL were loaded in a microfluidic array plate (MAP16), and filled with 15 μL of QuantStudio™ Absolute Q™ Isolation Buffer (Applied Biosystems™, Foster City, CA, USA). The thermal profile consisted of15 min of hot-start at 95 °C, followed by 40 cycles of denaturation at 95 °C for 5 s and subsequent annealing-extension at 64 °C for 30 s. The data were analyzed with QuantStudio™ Absolute Q™ Digital PCR Software v6.3.5 (Applied Biosystems™, Foster City, CA, USA).

##### 2.3.1.2 QX600

The first ddPCR machine included was the QX600™ AutoDG™ Droplet Digital™ PCR System. After droplet generation, the plates were sealed with a PX1 PCR Plate Sealer, and the amplification reactions were performed in a C1000 Touch Thermal Cycler. All machines were manufactured by Bio-Rad (Bio-Rad Laboratories, Inc., Hercules, CA, USA). The reactions were prepared considering a final reaction volume of 22 μL composed of 11 μL of ddPCR™ Supermix for Probes (No dUTP) (Bio-Rad Laboratories, Inc., Hercules, CA, USA), a final concentration of 900 nM primers and 250 probes for toxR, tdh, and ure, along with 200 nM of the IAC with 1000 copies of IAC DNA; and 3 μL of template DNA, the remaining volume was filled with nuclease-free water. The amplification consisted of 10 min at 95 °C, followed by 40 cycles of dissociation at 94 °C, and annealing-extension at 63 °C for 1 min. The reaction was concluded with a final step at 98 °C for 10 min and 4 °C indefinitely prior to droplet fluorescence reading. The results were analyzed with the software QX Manager Standard Edition 2.4.0.70 (Bio-Rad Laboratories, Inc., Hercules, CA, USA).

##### 2.3.1.3 QXC

The second ddPCR platform under evaluation was the QX Continuum™ Droplet Digital™ PCR System, recently launched by Bio-Rad (Bio-Rad Laboratories, Inc., Hercules, CA, USA). The same final primer and probe concentrations detailed for the QX600 were used, with the exception that the final reaction volume was 16 μL, and that the master mix selected was the QX Continuum™ ddPCR™ Supermix for Probes (Bio-Rad Laboratories, Inc., Hercules, CA, USA). Once the plate was loaded, it was sealed with a PX1 PCR Plate Sealer. The thermal profile consisted of a step at 50 °C, hot-start activation at 95 °C, 40 cycles of dissociation at 95 °C, and annealing-extension at 64 °C. The duration of each step was preconfigured and not disclosed by the manufacturer. The results were analyzed with the software QX Insight ddPCR Analysis Software version 1.0.

#### 2.3.2 Multiplex qPCR

The multiplex qPCR assay targeted three *V. parahaemolyticus* genes, namely *toxR*, *tdh*, and *ure*. The final primer concentrations were 200 nM for all three genes, whereas the probe concentrations were 200 nM for *toxR* and 150 nM for *tdh* and *ure*. The IAC was added at a final concentration of 100 nM, primers and probe with 1000 copies of IAC DNA. All of these were pre-mixed in a 10X format. The final volume of 20 μL was composed of 10 μL of SsoAdvanced™ Universal Probes Supermix (Bio-Rad Laboratories Inc., Hercules, CA, USA), 2 μL of 10X primer mix, 3 μL of template DNA, and 5 μL of nuclease-free water (Tiaris Biosciences, Cordoba, Spain). The reactions were run in a CFX Opus 96 Real-Time PCR System and analyzed with Bio-Rad CFX Maestro 2.3 Software v5.3.022.1030 (Bio-Rad Laboratories, Inc., Hercules, CA, USA). The thermal profile consisted of a hot-start step at 95 °C for 3 min, followed by 40 cycles of dissociation at 95 °C for 5 s and annealing-extension at 64 °C for 30 s, as previously described (qPCR optimization details are provided in supporting information).

#### 2.3.3 Real-time LAMP

For real-time LAMP, the Fast Master Mix ISO-004 (OptiGene, Horsham, UK) was selected. The final reaction volume was 25 μL, composed of 15 μL of master mix, 1 μL of *toxR* 25X primer mix (200 nM F3/B3, 1200 nM FIP/BIP, 400 nM LF/LB), 1 μL of IAC 25X primer mix (200 nM F3/B3, 800 nM FIP/BIP, 400 nM LF/LB), 10^6^ copies of IAC plasmid, 3 μL of template DNA, and the remaining volume was filled with nuclease-free water (Tiaris Biosciences, Cordoba, Spain). The reactions were incubated at 68 °C for 30 min, with fluorescence acquisition every 30 s. The amplification step was followed by melt curve analysis consisting of 95 °C for 1 s, 80 °C for 20 s, and heating again up to 95 °C, with fluorescence acquisition during the process. The reactions were run, and the data were analyzed, with the same equipment as for the multiplex qPCR detailed above.

#### 2.3.4 Colorimetric LAMP

The colorimetric LAMP assay was performed in a MyCycler™ Thermal Cycler System (Bio-Rad Laboratories Inc., Hercules, CA, USA). Considering that colorimetric reactions are end-point and do not discriminate targets, *toxR* and IAC were run in parallel reactions, thus having 1 μL of either 25X *toxR* or IAC primer mix. In the case of the latter, the plasmid was also added, along with 3 μL of template DNA and 12 μL of ISO-010RT-VIS (OptiGene, Horsham, UK). The reactions were incubated for 60 min. If needed to aid in the color interpretation, pictures were taken with an iPhone 16Pro, and the R values of five spots of each tube, the four corners along with the center of the reaction, were averaged after colorimetric measurement with the free App Pixel Picker 1.3.0.23, as previously described by Abalo et al. (2024).

### 2.4 Sample processing

From June 23rd to September 8th 2026, three mussel samples were processed every week until September 1st, and the week of the 8th two additional samples were gathered. These were analyzed following the ISO 21872-1 (ISO, 2017, p. 21872). Briefly, 25 g of mussel and liquor were mixed with 225 mL of ASPW, homogenized for 30 s, and incubated at 37 °C for 5 h. After this initial enrichment, 1 mL was taken for DNA extraction, and another one was transferred to 10 mL of fresh ASPW, which was further incubated for another 18 h. Once the second enrichment step was completed, 1 mL was also taken for DNA extraction, and plates of Thiosulfate Citrate Bile Salts Sucrose (TCBS, Condalab, Madrid, Spain), CHROMagar™ Vibrio (CAV, CHROMagar Microbiology, Paris, France), CRITERION™ HardyCHROM™ Vibrio (CHV, HARDY Diagnostics, Santa Maria, CA, USA), Vibrio Chromogenic Agar (VCA, Condalab, Madrid, Spain), and Chromatic™ Vibrio (CV, Liofilchem, Roseto degli Abruzzi, Italy) were streaked. The plates were incubated at 37 °C for 18 – 24 h, and screened for typical colonies. Whenever these were identified, a real-time colony-LAMP, targeting the *toxR* gene, was performed following the procedure previously described by Garrido-Maestu et al. (2020). To this end, the reaction conditions were the same described above, but the 3 μL of template DNA, the suspect colony was directly resuspended in the reaction mix (detailed in Section 2.3.3) using a sterile pipette tip, the heat needed to perform the amplification, 68 °C for 30 min, was enough to lyse the bacteria, release the DNA and perform the amplification.

### 2.5 Estimation of the limit of detection (LOD)

To estimate the LOD, the last version (v12) of the mathematical model described by Wilrich & Wilrich was applied (Wilrich and Wilrich, 2009). Briefly, 13 mussel samples were spiked with decreasing concentrations of a fresh culture of *V. parahaemolyticus*. The inoculation pattern consisted of two samples at the highest concentration, three in the intermediate and eight in the lowest, subdivided in two groups of four inoculated with different concentrations but both below 5 CFU. After the initial 5 h enrichment, the spiked samples were analyzed by the different techniques described above. After the final 18 h enrichment, these samples were analyzed by qPCR, real-time, and colorimetric LAMP to serve as molecular reference. The samples were also plated on the different media previously mentioned.

Mussels had to be sterilized, given the fact that they were positive for *V. parahaemolyticus*. Thus, in addition to spiking the pathogen, natural microbiota gathered from cockles, negative for *V. parahaemolyticus*, was also spiked. This step was performed as previously described by Rocha et al. (Rocha-Grandal et al., 2025).

## 3 Results

### 3.1 Determination of the LOD

The LOD was determined based on the results obtained from 13 samples spiked in the range of 15.4 and 1.2 CFU/25 g. The lower range was prioritized resulting in the following sample distribution: 2 samples spiked with 15.4 CFU, 3 samples with 7.4 CFU, 4 samples with 3.7 CFU, and another 4 samples inoculated with 1.2 CFU. It is important to keep in mind for the interpretation of the dPCR/ddPCR results the stringent cut-off set, 5 times more positive partitions than the NTC, as this may impact the results.

#### 3.1.1 LOD after 5 h enrichment

**Fig. 1:**
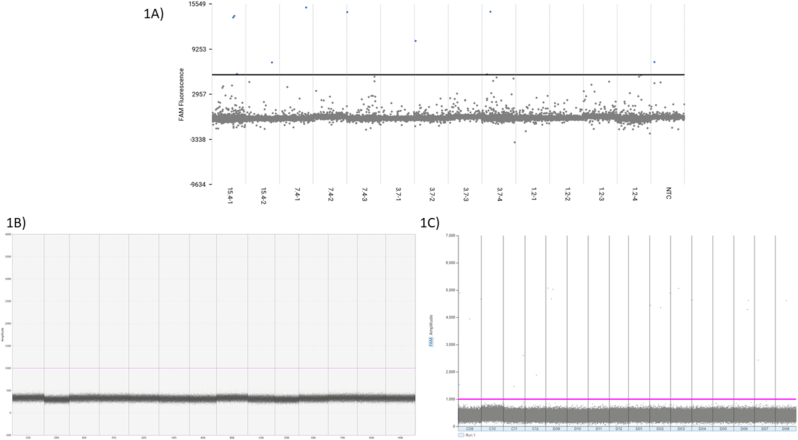

After the initial 5 h of enrichment, all samples were negative by dPCR/ddPCR regardless of the concentration level, and the machine was selected considering the positivity criterion established (see Fig. 1A, B, and 1C). Likewise, all samples were negative for LAMP, regardless of the detection strategy. When analyzing the results of qPCR, late amplifications (Cq > 38) were obtained with spiking levels of 7.4 and 3.7 CFU, respectively. Considering these results, the mathematical model determined an LOD50 of 15.0 CFU/25 g for qPCR (upper and lower 95 % confidence limits were 4.4 and 50.9 CFU/25 g respectively).

When focusing in the values obtained for dPCR/ddPCR, to what was previously mentioned, if a less stringent cut-off was set for calling a positive sample, e.g. 2-3 partitions, the reported LOD50 values would be significantly impacted, as they would now be in the ranges of 4.5 – 21.6, 21.6, and 3.0 – 6.2 CFU/25 g for the AbsQ, QX, and QXC, respectively. It is important to keep in mind that specific workflows are being compared (different number of targets and detection chemistries) which may impact the final outcome.

#### 3.1.2 LOD after 24 h enrichment

When the analyses were performed after the secondary enrichment, a higher number of positive samples were obtained, with identical results for all three molecular techniques, resulting in an LOD50 of 3.3 CFU/25 g (upper and lower 95 % confidence limits were 1.4 and 7.7 CFU/25 g, respectively). The results obtained after plating in the different chromogenic media are summarized in Table 2.

**Table 1:** Primers and probes used for LAMP, qPCR and dPCR.

Technique	Primer	Sequence (5′ → 3′)	Final concentration (nM)	Reference
LAMP_toxR	F3	TTG AAG TTG AAG AGC CAG CT	200	This study
	B3	GCA GTA CGC AAA TCG GTA GT	200	
	FIP	GAA GTC GTC GCT AAA GAC GGC T *tttt* AAC AAT GAC GCC TCT GCT AA	1200	
	BIP	CGC AAT CGT TGA ACC AGA AGC G *tttt* AAC GCG TGG AAT CCA AGG	1200	
	LF	TGA GGT AGA AAC GAT CGT AG	400	
	LB	AGC ACC TGT GGC TTC TGC TG	400	
LAMP_IAC	F3	ACC TTT CCT CCC CTT GAG TC	200	Rocha-Grandal et al. (2025)
	B3	CGA TGG ACA CGG TTT GTT GA	200	
	FIP	GCG TAT TCG TCC GTT GGA CCT C *tttt* CGC TCC TTA GGG TCG TCT	800	
	BIP	CCG AGG CTG CGG CAA GCA A *tttt* GCG GCC TAG CGT AGC AA	800	
	LF	TAC TGT GCC GCA CAG CG	400	
	LB	GGT GGG AGT CTC TAG GAG CCA	400	
dPCR/qPCR^a^	toxR_P3F2	TAG CGA CGA CTT CTG ACG CA	200	This study
	toxR_P3R2-2	AAA CAG CAG TAC GCA AAT CGG TAG TAA T	200	
	toxR_P3P2	^FAM^– AGC ACC TGT/^ZEN^/GGC TTC TGC TGT GAA TCC – ^IABkFQ^	200	
	tdh-P3F1	CCA TGT TGG CTG CAT TCA AAA C	200	
	tdh-P3R1	CGG TTT GTC CAA AAG TCA GAG AC	200	
	tdh2-P3P	^TxR^®^−X NHS^ – TCT GTC CCT TTT CCT GCC CCC GGT – ^IAbRQSp^	150	
	ureR-P3F4	TGA TAA CCC AGG ATC CAC AAA GAA A	200	
	ureR-P3R4	CCG ATA ACT GAT CTG AGA AAC GGT	200	
	ure2-P3P	^Cy^®^5^ – CCA CCC TTC/^TAO^™/GCC GAC ATC TGC GC – ^IAbRQSp^	150	
	IAC-F	AGT TGC ACA CAG TTA GTT CGA G	100	Garrido-Maestu et al. (2018)
	IAC-R	TGG AGT GCT GGA CGA TTT GAA G	100	
	IAC-P	^YY^ – AGT GGC GGT/^ZEN^™/GAC ACT GTT GAC CT – ^IABkFQ^	100	(Garrido-Maestu et al., 2019)

a 1000 Copies of NC-IAC DNA were added as control. YY: Yakima Yellow is a trademark from IDT. “*tttt*” is poly-T linker between F2-F1c and B2-B1c.

**Table 2:** Selective solid media comparison.

Type of sample	N	TCBS		CAV		CHV^a^		VCA^a^		VC^a^	
		Typical	Confirmed (%)	Typical	Confirmed (%)	Typical	Confirmed (%)	Typical	Confirmed (%)	Typical	Confirmed (%)
Mussels	29	2	2 (100)	6	6 (100)	26	1 (4)	7	4 (57)	4	4 (100)
LOD	15	7	7 (100)	7	7 (100)	7	7 (100)	7	7 (100)	7	7 (100)

a Data recorded from 26 samples. N: total number of samples analyzed. TCBS: Thiosulfate Citrate Bile salts Sucrose. CAV: CHROMagar™ Vibrio. CHV: CRITERION™ HardyCHROM™ Vibrio. VCA: Vibrio Chromogenic Agar. CV: Chromatic™ Vibrio.

### 3.2 Samples

A total of 34 natural, non-spiked, and non-depurated mussel samples were analyzed by the different techniques. All of them were gathered in the region of the Rias Baixas in Galicia, northwestern Spain. The distribution was as follows: 50 % from Arousa (17 samples), 38.2 % from Vigo (13 samples), and 11.8 % from Pontevedra (4 samples). These Rias cover the majority of the mussel production in the region (FAO, 2026).

#### 3.2.1 Species identification

**Fig. 2:**
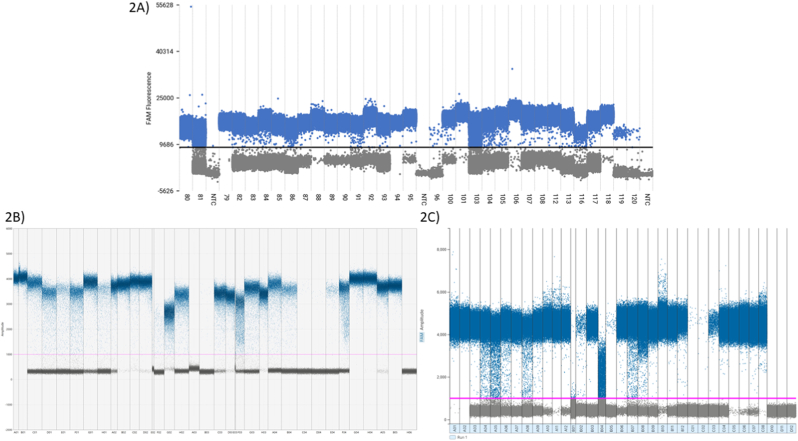

After 5 h of enrichment, identical results were obtained with the dPCR approaches (AbsQ, QX, and QXC) and qPCR (32 samples were analyzed by QXC); all samples were positive except for one due to an “image registration error” in the AbsQ machine (see Fig. 2A, B, and 2C). Minor discrepancies were recorded with both LAMP detection strategies, as only 2 samples were negative. In a similar way, once the two-step enrichment process, detailed by the ISO standard, was completed and analyzed, all samples were positive by qPCR, real-time LAMP, and colorimetric LAMP.

#### 3.2.2 Molecular characterization

**Fig. 3:**
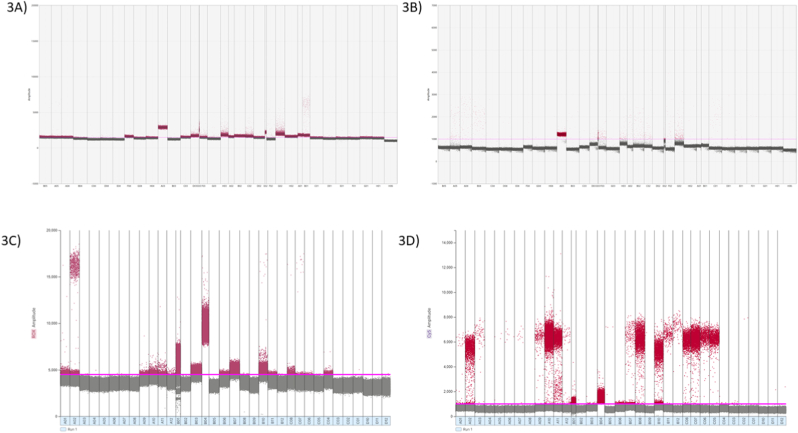

Due to fluorophore compatibility, it was only possible to sub-characterize the samples according to the presence of virulence genes, by QX, QXC, and qPCR. Important differences were observed depending on the platform selected, as with QX 94.1 % of the samples were identified as *tdh*-positive, but only 65.6 % by QXC and 32.4 by qPCR. Likewise, when analyzing the *ure* gene, 61.8 % were positive by QX, while 75 % were positive by QCX, and 55.9 % by qPCR. The results for the double positives, *tdh*/*ure*, were similar among ddPCR platforms, as 58.8 % were positive with QX and 62.5 % by QCX, with the qPCR reporting the lowest percentage of double positives with a 26.5 % (see Fig. 3A–D).

#### 3.2.3 Culture confirmation

After the secondary enrichment, 31 out of the 34 samples were plated on five *Vibrio*-specific solid media, namely TCBS and four chromogenic agars. The worst results in terms of specificity were recorded with CHV, as all samples produced typical colonies, but it was only possible to confirm the presence of *V. parahaemolyticus* in 1 of them. Regarding the other media, 6 samples presented typical colonies on CAV, 3 on CV, which were confirmed, and 7 samples had typical colonies on VCA, but only 4 were confirmed. These results are summarized in Table 2.

## 4 Discussion

Rapid detection of foodborne pathogenic microorganisms is an important research field due to the implications it poses for consumers and producers (Aladhadh, 2023; Panwar et al., 2022). his becomes particularly relevant when dealing with food commodities with short shelf lives, such as seafood (Anagnostopoulos et al., 2022). In this specific type of foods, *Vibrio* spp. are well-known human pathogens, more specifically *V. cholerae*, *V. parahaemolyticus*, and *V. vulnificus* (Brauge et al., 2024). The advent of molecular methodologies, particularly PCR/qPCR, has allowed for a significant reduction in the turnaround times of classical microbiological methods, from around one week to just 24 – 48 h, as reported in previous studies (Blackstone et al., 2007; Fedio et al., 2007; Nordstrom et al., 2007). However, additional efforts are required to accelerate the current methodologies. This task was approached in the present work, taking advantage of the dPCR and ddPCR techniques, both of which perform reaction partitioning but differ in the way they do so; the first does it with a special plate that has thousands of microchambers, while the second generates thousands of droplets with a special oil. These approaches have been reported to be more sensitive than existing technologies (Huggett et al., 2013; Kokkoris et al., 2021; Vogelstein and Kinzler, 1999). The assay under study targeted *V. parahaemolyticus*; it was implemented after the initial enrichment step indicated in the ISO standard for this pathogen (ISO, 2017), and it was side-by-side compared to qPCR and LAMP. For confirmation purposes, the ISO standard was completely followed, the enriched samples were analyzed by qPCR and LAMP, and plated in different solid media to evaluate existing options of commercial media, considering the issues reported to isolate *V. parahaemolyticus* in certain types of samples (Rocha-Grandal et al., 2025).

**Fig. 4:**
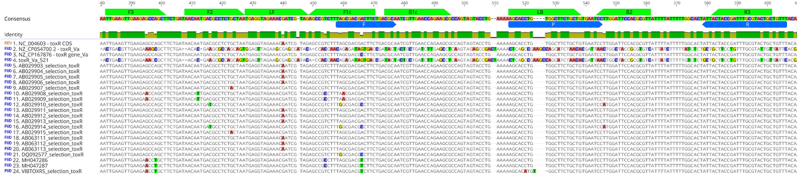

To be applied in the present work, new primers and probes were designed for implementation in dPCR, ddPCR, qPCR, and LAMP assays. This was motivated by the identification of a region within the *toxR* gene with higher genetic variability between *V. parahaemolyticus* and *V. alginolyticus*, see Fig. 4. In the particular case of qPCR, *tdh* and *ure* genes, primers, and probes were also included. The *ure* gene was included as a surrogate for the *trh* gene (Nilsson and Turner, 2016).

In order to assess the potential of dPCR/ddPCR for the rapid detection of *V. parahaemolyticus* after the first enrichment step, samples spiked in the range of 1.2 to 15. 4 CFU/25 g were prepared. Considering that no universal consensus exists in regards to the minimum number of positive partitions to call a positive result, it is important to keep in mind that a stringent cut-off was set in the present work to ensure maximum specificity, and to avoid calling false positive samples. At this stage, no positive samples were classified; however, this could be related to the stringent cut-off selected, as even though no positive calls were considered, there were actually positive partitions in all platforms (see Table S1). Previous works have indicated that only 2-3 partitions are enough for a positive call (European Commission. Joint Research Centre, 2019; Gatto et al., 2022), to consider the amplitude of the fluorescence (Kokkoris et al., 2021), or to consider three times more partitions than the negative control, instead of five (Bio-Rad, 2018). If these criteria were applied, the LOD50 values would be in the ranges of 4.5 – 21.6, 21.6, and 3.0 – 6.2 CFU/25 g for the AbsQ, QX, and QXC, respectively. When the LOD samples were analyzed by qPCR, it was important to note that, even though positive values were obtained, these presented a very high Cq value. The fact that such high LOD50 values and confidence intervals were obtained indicates that additional replicates are needed in order to achieve a more accurate LOD determination after 5 h of enrichment (LaBudde and Wehling, 2023). When these samples were analyzed by LAMP, all were negative regardless of the detection strategy selected, which is in agreement with D'Agostino et al., who indicated that, regardless of what has been extensively published, LAMP was not more sensitive than qPCR when analyzing *Salmonella* spp. (D'Agostino et al., 2015). This was also reported with other bacterial and viral pathogens in recent years (Garrido-Maestu et al., 2018; Y. Wang et al., 2015; Zhang et al., 2011). These results stress the importance of appropriate sample processing, a topic which tends to be overseen, as highlighted by Brehm-Stecher et al. and other authors (Brehm Stecher et al., 2009). Without proper processing, particularly in food science, where pathogens tend to be present at low concentrations, the success of the most sensitive and late-generation detection technologies is hindered (Dwivedi and Jaykus, 2011). Future work, focusing on enrichment broth modifications, incubation temperature, and dilution factor, among other factors, may enable same-day detection of *V. parahaemolyticus* in a similar way to what was achieved by previous authors for *Salmonella* spp., *L. monocytogenes,* and/or Shiga Toxin-producing *E. coli* (Azinheiro et al., 2022; Fachmann et al., 2017; Garrido-Maestu et al., 2020; Löfström et al., 2009). It may be noted from these studies that the short enrichment step is a key factor which may change among different bacteria, e.g. *L. monocytogenes* required a slightly longer incubation. A consistent approach among all of them relied in the reduction of the dilution factor which allowed, on the one side, to speedup the heating processing thus reaching the optimal growth temperature faster; and on the other hand, to recover virtually all the broth for DNA extraction and plate confirmation. One other key factor, which may further allow to improve the current results, may be the replacement of the DNA extraction protocol for a more efficient one, such as the lysis-GuSCN from Kawasaki et al. (2005), the glassmilk (Costa-Ribeiro et al., 2024; Page et al., 2022) or even commercial kits like the commonly used DNeasy series (Elizaquivel and Aznar, 2008; Papatheodorou et al., 2021) however, careful evaluation must be made in order to assess the tradeoff between cost and turnaround time.

The secondary enrichments were not analyzed by dPCR, neither from the LOD nor from the natural samples. This is a limitation in the current study however, it was motivated by the fact that the goal of the study was to take advantage of the higher sensitivity of dPCR/ddPCR over qPCR and LAMP, to detect *V. parahaemolyticus* after only 5 h. If the second enrichment was needed, considering the cost/benefit aspect, it would not be worth pursuing this technique instead of other more economical ones providing the same results (Junjie, 2020; W, 2024).

When the determination of the LOD was applied after the secondary enrichment step, as expected, the LOD50 was significantly reduced. This was particularly evident for LAMP assays, as after 5 h, all samples were negative, but once the secondary enrichment was analyzed, it was possible to reach an LOD50 of 3.3 CFU/25 g, the same as for qPCR. This improvement in the LOD50 values was motivated by the fact that the extended enrichment step compensates for the lower sensitivity of LAMP, in a similar way to what D'Agostino et al. reported for the detection of *Salmonella* spp., highlighting once again the importance of appropriate sample processing and the current utility of bacterial enrichment (D'Agostino et al., 2015; Law et al., 2015). All positive qPCR and LAMP sample results were confirmed by the appearance of typical colonies on all selective media tested. These colonies were confirmed by the colony LAMP assay, verifying the suitability of this strategy for rapid isolate confirmation, as previously described for *L. monocytogenes* (Garrido-Maestu et al., 2020). Overall, these results support the fact that after appropriate enrichment, LAMP, regardless of the detection strategy, may be reliably used for the detection of this and other pathogens, in decentralized, low-resource setups.

When moving the analysis to natural, non-spiked, non-depurated mussel samples, it was worth noting that all 34 samples collected were positive by dPCR/ddPCR and qPCR, and 32 by the LAMP approaches. These results can be explained by the fact that, given the season when the mussels were collected, summer in the northern hemisphere, a high prevalence of *V. parahaemolyticus* was present in the waters where these mollusks were cultivated (Garrido-Maestu et al., 2016; Martinez-Urtaza et al., 2008). Important differences were observed among the number of positive samples for each of the virulence genes studied; in this sense, the QX reported a higher incidence of *tdh*-positive samples compared to *ure* (94.1 % vs 61.8 %), while with the QXC the number of *ure-*positive was higher (75 % vs 65.6 %) being these results similar to those reported in previous studies performed on the area (Garrido-Maestu et al., 2016). The results were comparable among platforms when comparing the number of double-positive samples, *tdh*/*ure*, as 58.8 % were identified with the QX and 62.5 % with the QXC. Higher discrepancies were obtained when comparing ddPCR with the qPCR, where 32.4 % of the samples were positive for *tdh,* 55*.9* % for *ure*, and 26.5 % for both. These values decreased to 26.5 % and 8.8 % for individual genes, and double positive when the analyses were performed in the 24 h enriched samples. These discrepancies may be explained by platform performance, thus results may be taken cautiously as, given the fact that not all the machines had the exact same setup, this may be affecting the amplification performance of each of the genes within the reaction; it may be noted that the *toxR* exhibited early, and strong, amplification already after 5 h of enrichment, and even more after 24 h, that may outcompete the other targets, and deplete the reagents of the reaction. However, additional data may be needed to further support this hypothesis. Due to reaction partitioning, this phenomenon does not impact dPCR/ddPCR assays as much (Gaňová et al., 2021). It is important to note that the number of targets detected with the different technologies was not even, thus the impact of target multiplexing must be kept in mind.

The final part of this study presented herein was focused on comparing different chromogenic agars for the selective isolation of *V. parahaemolyticus*. This comparison among media was motivated by the reported problems related to the isolation of *V. parahaemolyticus* (Blanco-Abad et al., 2009; Earle and Crisley, 1975; Richards et al., 2012; Rocha-Grandal et al., 2025). Even though previous studies have reported good results when using ChromID™ Vibrio from BioMerieux (BioMérieux S.A., France), this medium was discarded given the fact that it is only sold as ready-to-use plates, thus reducing its shelf life and overall applicability (Bonnin-Jusserand et al., 2019; Garrido et al., 2012). Vibrio Chromoselect Agar from Merck (Chromoselect, Merck Millipore, Burlington, MA, USA), and HiCrome™ Vibrio (HC, Sigma–Aldrich, St. Louis, USA), despite their availability as a dehydrated form, were also discarded based on previous studies (Garrido-Maestu et al., 2014a, Garrido-Maestu et al., 2014b; Rocha-Grandal et al., 2025). In the present work, it was observed that all the media under evaluation allowed the isolation of *V. parahaemolyticus* from the set of spiked samples prepared for the determination of the LOD. One subjective observation in this set of samples was that VCA, from CONDA, had a darker, yellowish background, thus making it a bit more challenging to differentiate typical mauve colonies. In this line, when streaking on CV, from Liofilchem, the color intensity of the typical colonies seemed less intense compared to the other agars, thus also making typical colony screening a bit more cumbersome. Even though CHV seemed like a promising option due to its potential applicability to further discriminate among *V. cholerae* and *V. vulnificus* (https://hardydiagnostics.com/media/assets/product/documents/CRITN-HardyCHROMVibrio.pdf), not possible with any of the other media, it was observed that this agar had low specificity, as when it was used with non-spiked samples, in most cases typical colonies were observed, however, these were not confirmed as *V. parahaemolyticus*. These discrepancies among spiked and non-spiked results, even though in all cases strong molecular signal for *V. parahaemolyticus* was observed, were explained by overgrowth of other microbes, mainly *V. alginolyticus* (Lee et al., 2020; Yeung and Thorsen, 2016) which was actually reported to be highly prevalent in the region under study (Gómez-León et al., 2005; Hidalgo et al., 2008), even representing more than 90 % of suspect isolates in previous works (Martinez-Urtaza et al., 2008). This confounding similar morphology was the same issue reported for HiCrome Vibrio and Vibrio Chromoselect Agar (Garrido-Maestu et al., 2014; Rocha-Grandal et al., 2025). Additionally, the problematic reported with *V. alginolyticus* would be in agreement with what was observed on CAV, where non-spiked samples mainly presented white colonies, which are typical of *V. alginolyticus* (Rocha-Grandal et al., 2025). In addition to this, other factors such as entrance in viable but nonculturable state (VBNC) (Zhao et al., 2020), matrix effect on the different agar media (Yeung and Thorsen, 2016), among others (Banerjee and Farber, 2017; Yoon and Lee, 2022)must to be overseen as they, individually, combined, or along with *V. alginoloyticus* may be behind these discrepancies. Taken together, CAV reported the best results to be used as a complement of the mandatory medium, TCBS, as it allowed isolating and confirming a higher number of *V. parahaemolyticus*-positive samples with higher specificity, as summarized in Table 2. This concurs with previous studies (Lee et al., 2020; Pinto et al., 2011) and reinforces the utility of this particular combination TCBS/CAV (Nigro and Steward, 2015).

## 5 Conclusion

The results obtained in the present study support the implementation of different DNA amplification techniques, namely dPCR/ddPCR, qPCR, and LAMP, for the rapid detection of *V. parahaemolyticus* in mussel samples. Although dPCR/ddPCR has been reported to be more sensitive than other nucleic acid amplification techniques, without appropriate sample preparation/processing, it is not possible to improve the detectability of this pathogen. Moreover, no differences were observed in qPCR, and only minor differences were observed in real-time LAMP and colorimetric LAMP, indicating that after appropriate enrichment, LAMP may be used reliably in decentralized, low-resource setups, such as mussel rafts. Finally, CAV remained the best chromogenic agar option to be used along with TCBS.

## CRediT author statement

Ana Costa-Ribeiro: investigation and revision.

David Rocha-Grandal: investigation and revision.

Lara Pierantoni: investigation and revision.

Carlos Honrado: investigation and revision.

Lorena Diéguez: investigation, funding acquisition, and revision.

Alexandre Lamas: supervision, validation and revision.

Alejandro Garrido-Maestu: conceptualization, methodology, supervision, validation, funding acquisition, writing of the original draft.

## Declaration of competing interest

I hereby declare on behalf of myself, and my co-authors, that the we do not have any conflict of interest to declare in regards to the present study.
