# Supplementary material for: Application and evaluation of digital PCR platforms for same-day detection of Vibrio parahaemolyticus in mussel samples
Source: Curr Res Food Sci. 2026 Jun 23;13:101484. doi: 10.1016/j.crfs.2026.101484 (PMC13316628; doi:10.1016/j.crfs.2026.101484)
Supplement: Multimedia component 1 [file mmc1.docx]

**Supporting Information**

**Application and evaluation of digital PCR platforms for same-day detection of *Vibrio parahaemolyticus* in mussel samples**

Ana Costa-Ribeiro^1,2^, David Rocha-Grandal^1,3^, Lara Pierantoni^4^, Carlos Honrado^4^, Lorena Diéguez^4^, Alexandre Lamas^5^, and Alejandro Garrido-Maestu^1*^

^1^Laboratory of Microbiology and Technology of Marine Products (MicroTEC), Institute of Marine Research (IIM), CSIC, Eduardo Cabello, 6, 36208, Vigo, Spain

^2^Department of Biochemistry, Genetics and Immunology, University of Vigo, 36310 Vigo, Spain

^3^Department of Functional Biology and Health Sciences, University of Vigo, 36310 Vigo, Spain

^4^Medical Devices Research Group, International Iberian Nanotechnology Laboratory, Avenida Mestre José Veiga s/n, 4715-330 Braga, Portugal

^5^Food Hygiene, Inspection and Control Laboratory (LHICA), Department of Analytical Chemistry, Nutrition and Bromatology, Veterinary School, Campus Terra, Universidade de Santiago de Compostela 27002, Lugo, Spain

*Correspondence: [agarrido@iim.csic.es](mailto:agarrido@iim.csic.es)

1. Primer design
   1. qPCR

All primers and probes were designed with Primer3 Plus (Andreas Untergasser Xiangyu Rao, Ton Bisseling, Ren Geurts, and Jack A.M. Leunissen, 2007). As reference sequence, for *toxR*, sequences of *V. parahaemolyticus* and *V. alginolyticus*, species known to interfere with *V. parahaemolyticus* detection, were retrieved from NCBI, and aligned taking advantage of Genenious Prime® (Geneious Prime® version 2026.1.1. (Biomatters Ltd., Auckland, New Zealand), see Figure S1.

The desing of the primers and probe for the *tdh* and *ure* genes was done as previously described but using sequence NC_004605 and CP006005 as reference sequences respectively.

- 1. LAMP

The same procedure to generate the consensus sequence detailed in 1.1 was followed, but the design was performed in Primer Explorer V5 (<https://primerexplorer.jp/e/index.html>). See Figure S1.

1. qPCR Assay optimization

The same procedure was followed for all three genes (*toxR*, *tdh* and *ure*).

- 1. Primer and probe optimization

Primer concentration was optimized from 200 to 800 nM. Even though faster amplification was observed at higher concentrations, these also generated unspecific amplifications thus 200 nM was selected. See figures S2A and S2B.

For probe optimization, the concentrations tested were 150 nM, 200 nM and 250 nM, being 200 nM determined to be optimal. See Figure S2C.

- 1. Temperature optimization

Once the optimal concnetrations were determined, the optimal amplification temperature was determined testing the range 62, 64 and 66 °C, being 64 °C determined to be the optimal. See Figure S2D. For *tdh* and *ure* the optimal temperature determined for *toxR* was used.

- 1. Inclusivity and exclusivity test

For *toxR* a panel of 8 different *V. parahaemolyticus* strains was used to confirm inclusivity, while 18 non- *V. parahaemolyticus* were used for exclusivity. The exclusivity included 3 *V. vulnificus*, 2 *V. cholerae*, 1 *V. mimicus*, 1 *V. anguillarum*, 2 *V. alginolyticus*, 2 *Salmonella* spp., 1 *Enterococcus* spp., 1 *Staphylococcus* spp., 1 *Pseudomonas* spp., 2 *L. monocytogenes* and 2 *E. coli*.

Regarding *tdh* and *ure*, 2 and 3 *V. parahaemolyticus* positive strains were used for inclusivity respectively, which also served for exclusivity evaluation.

- 1. Dynamic range and amplification efficiency in simplex and multiplex formats

Performing ten-fold serial dilutions of pure DNA it was possible to cover a 7 log range in simplex assays for *toxR, tdh* and *ure*, from 3.4 ng/ µL down to 0.00034 pg/ µL for *toxR* and *ure*, while for *tdh* was from 6.72 ng/ µL down to 0.00067 pg/ µL. When multiplexing assays 6 logs were covered, from 27.2 ng/ µL down to 0.027 pg/ µL.

1. LAMP assay optimization

The LAMP assay optimization was performed based on the procedure previously described by Roumani et al. (Roumani et al., 2021).

- 1. Primer optimization

The outer primers F3/B3 were fixed as 200 nM and the concentration range from 600 to 1200 nM of FIP/ BIP was analyzed. From this experiment 1200 nM was determined to be optimal and the range of 200 to 800 nM of LF/ LB was tested, being 400 nM stablished as the optimal. With these final concentrations, 200 nM for F3/B3, 1200 nM FIP/BIP and 400 nM for LF/LB the addition of supplements was tested. See figures S3A to S3C

- 1. Supplement evaluation

The addition of pullulan was reported to enhance specificity of LAMP assays (Gao et al., 2019), thus the supplementation of the reaction with 1% was assess, however, no enhancing effect was observed thus it was not added to the final assay. See Figure S3D.

- 1. Temperature optimization

The last step consisted in the optimization of the temperature. For this step, a gradient from 60 to 70 °C was tested, being 68 °C determined to be the best condition. See Figure S3E.

- 1. Inclusivity and exclusivity

For the inclusivity panel a total of 10 *V. parahaemolyticus* strains were tested, and for the exclusivity 15 non- *V. parahaemolyticus* were analyzed, including 3 *V. vulnificus*, 1 *V. cholerae*, 1 *V. mimicus*, , 1 *V. alginolyticus*, 2 *Salmonella* spp., 1 *Enterococcus* spp., 1 *Staphylococcus* spp., 1 *Pseudomonas* spp., 2 *L. monocytogenes* and 2 *E. coli*.

- 1. Dynamic range

The *toxR* simplex dynamic range covered 6 logs, from 3.4 ng/ µL down to 0.0034 pg/ µL, while the multiplex with the IAC covered 4 logs, 3.4 ng/ µL down to 0.34 pg/ µL.


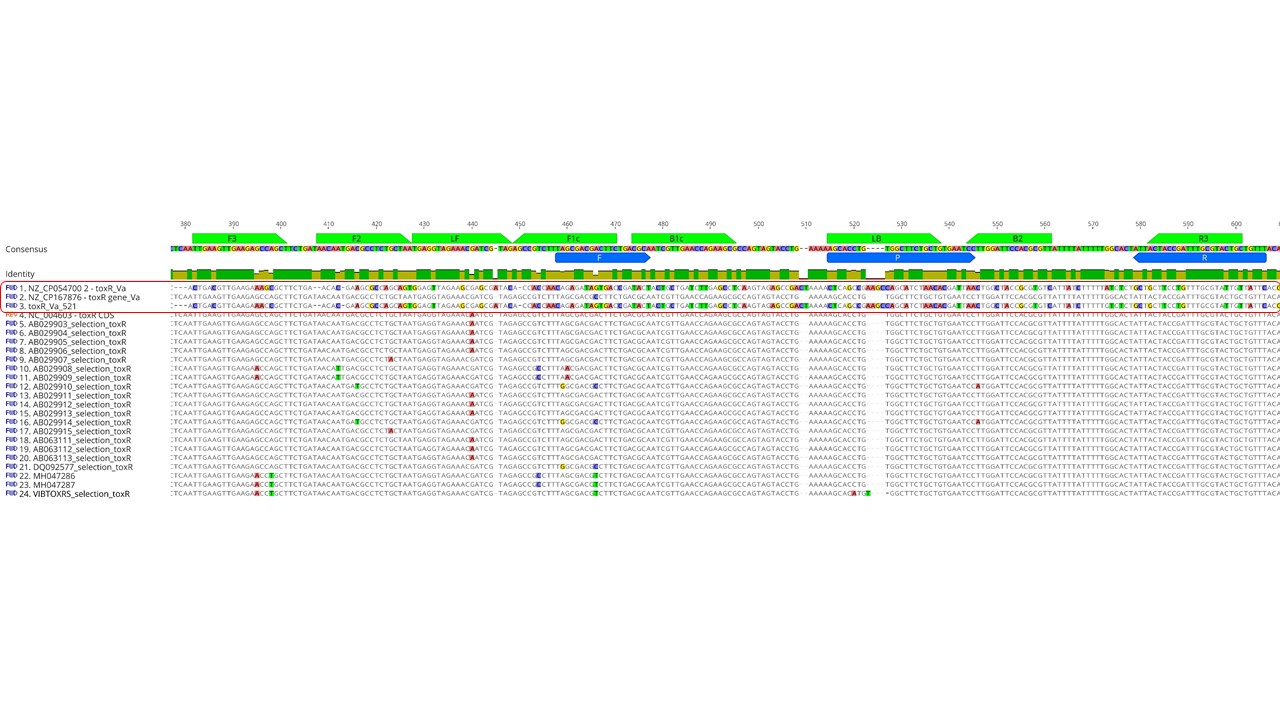


Figure S1. Sequence alignment generated for *toxR*. Highlighted in the red box the sequences from *V. alginolyticus* are provided. In green, the position of LAMP primers is indicated, and in blue qPCR primers and probe are indicated.


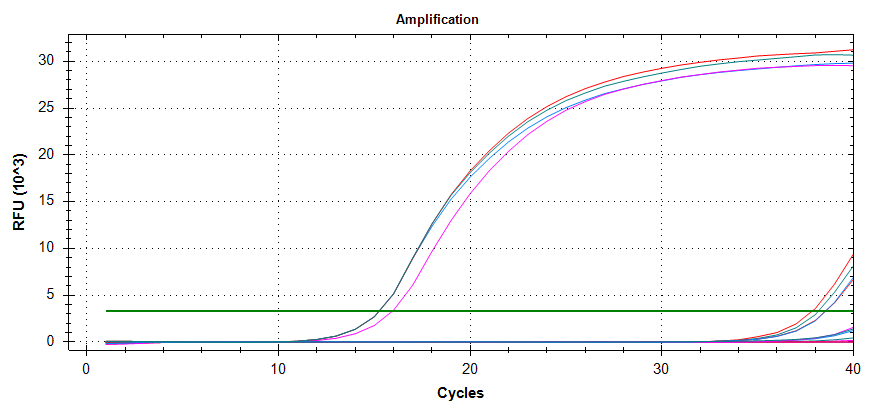


A)


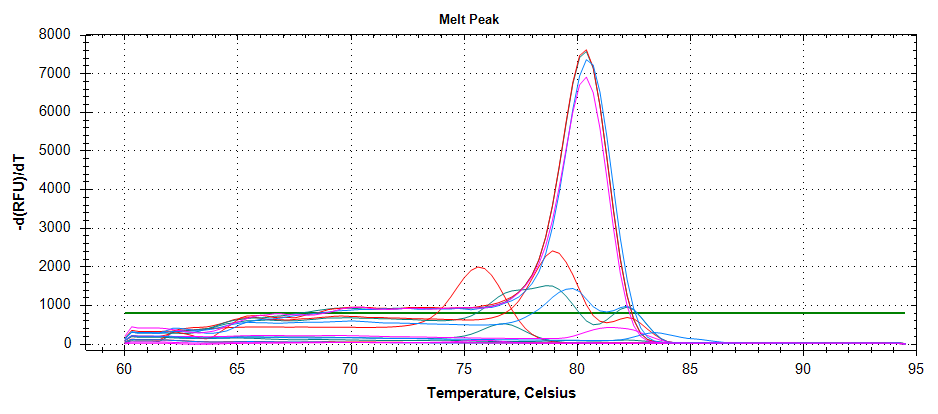


B)


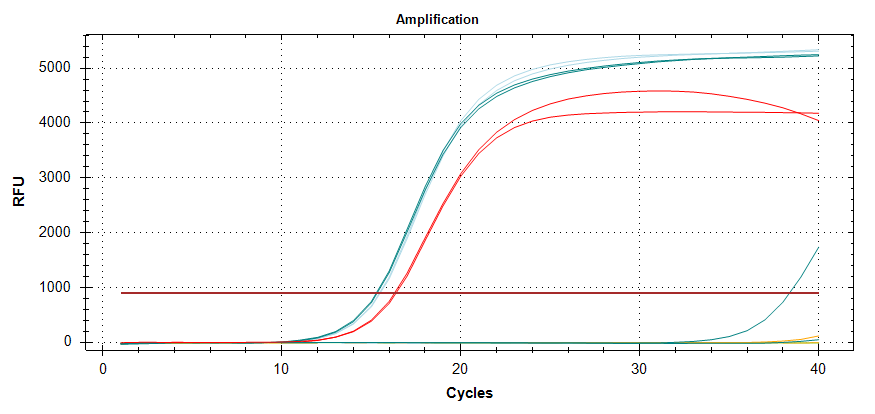


C)


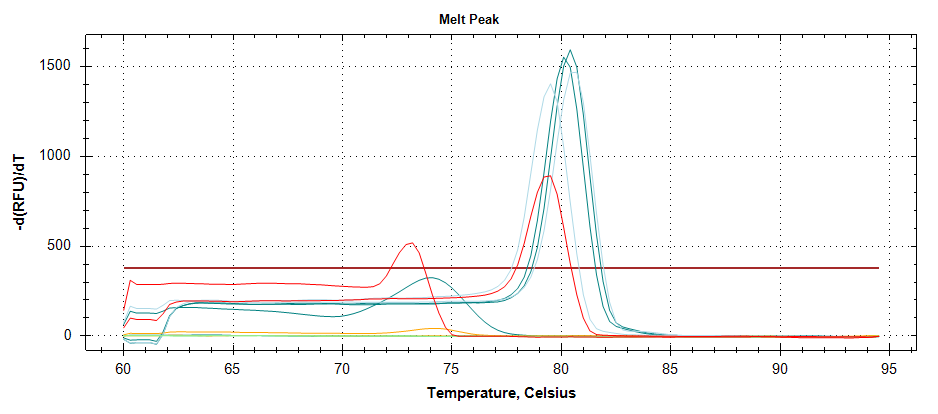


D)


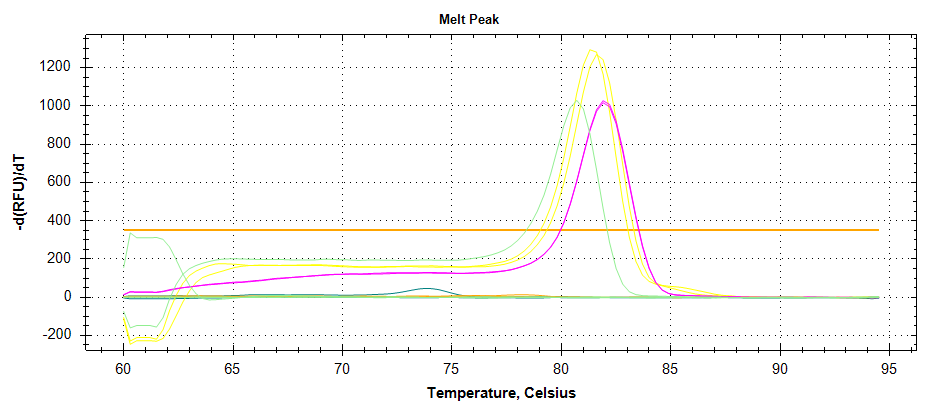


F)


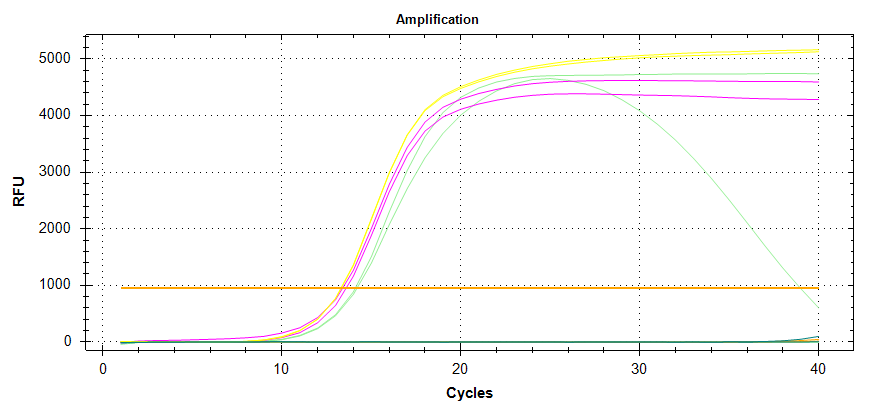


E)

G)


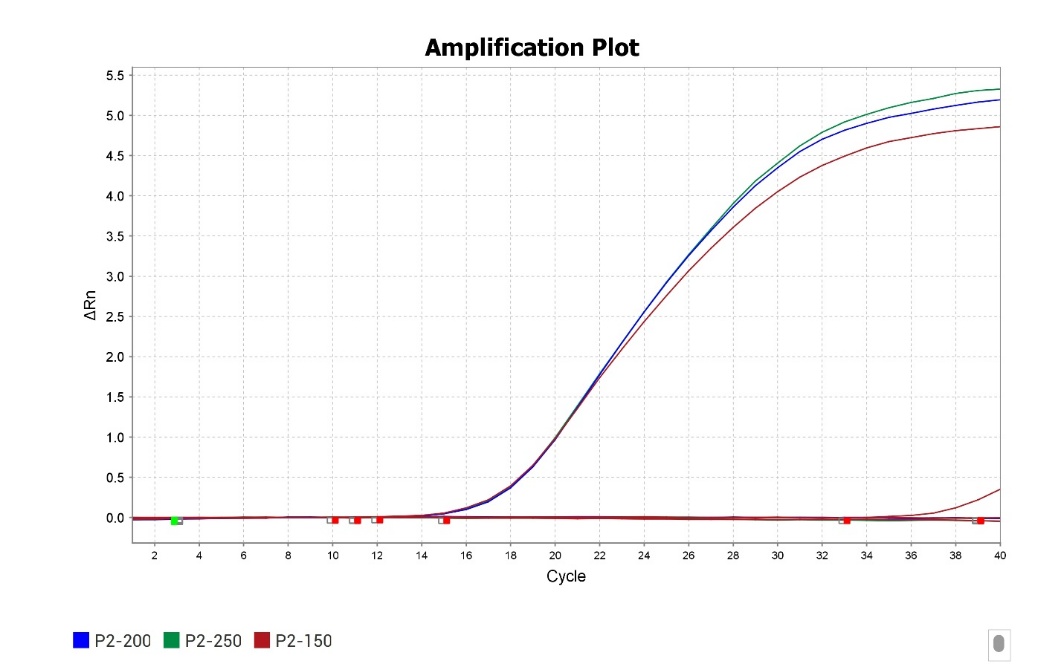


J)


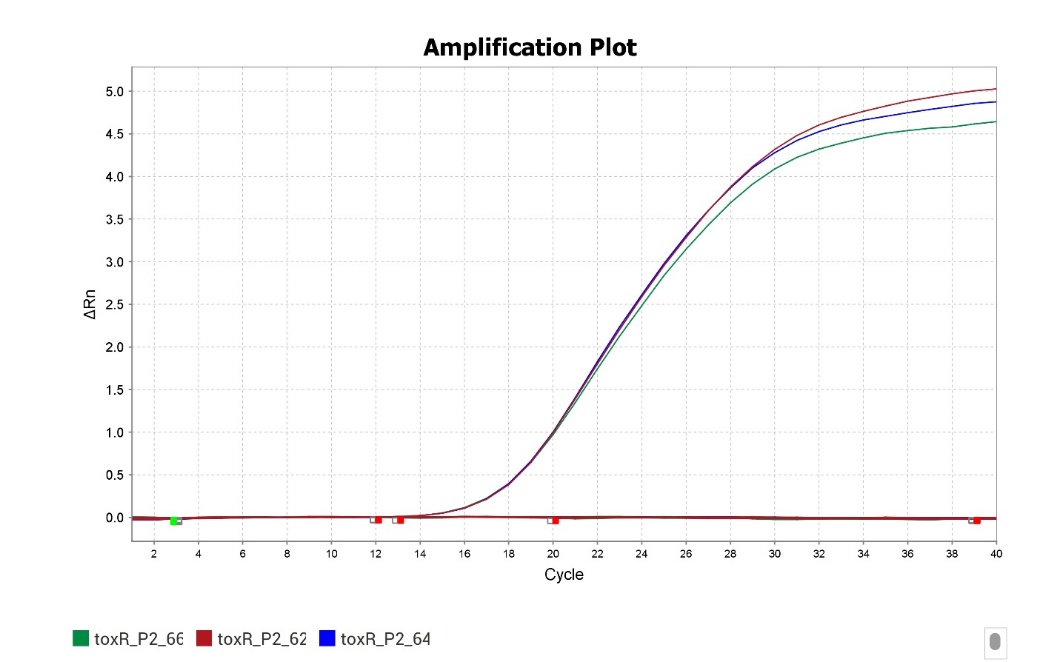

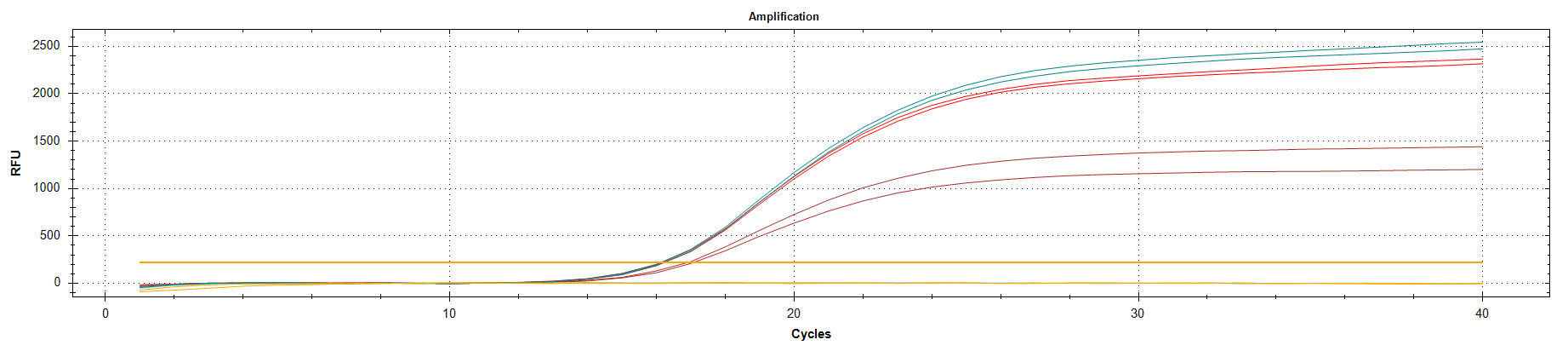


I)


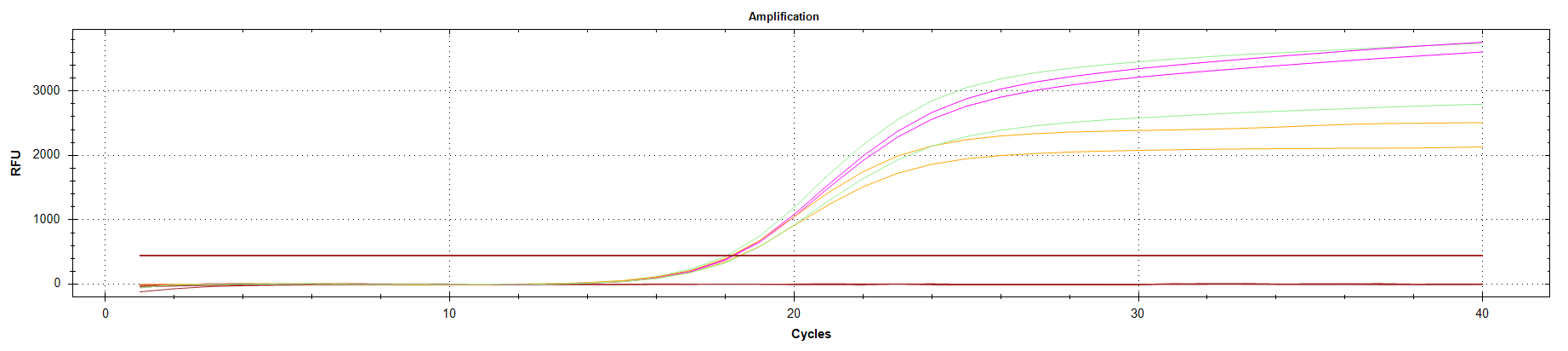


H)


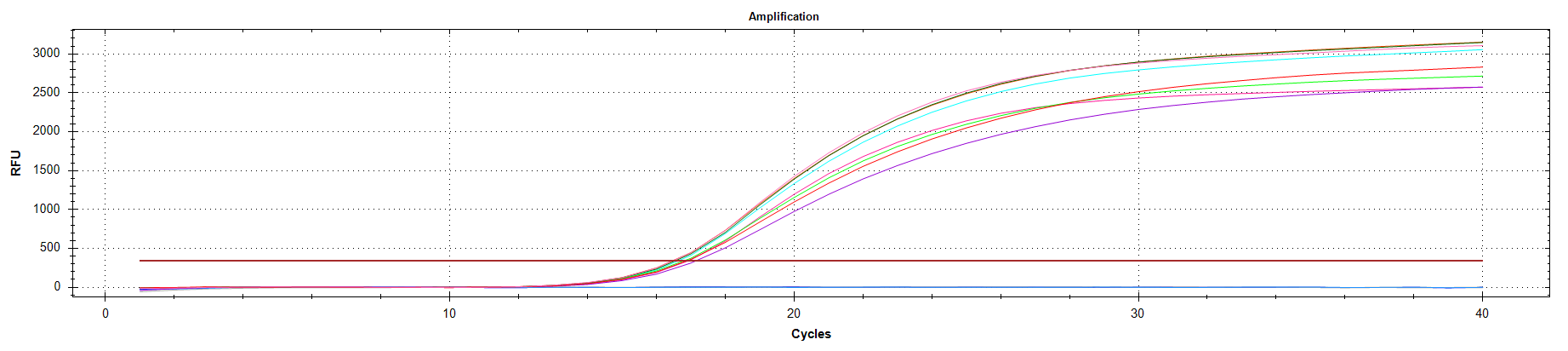


L)


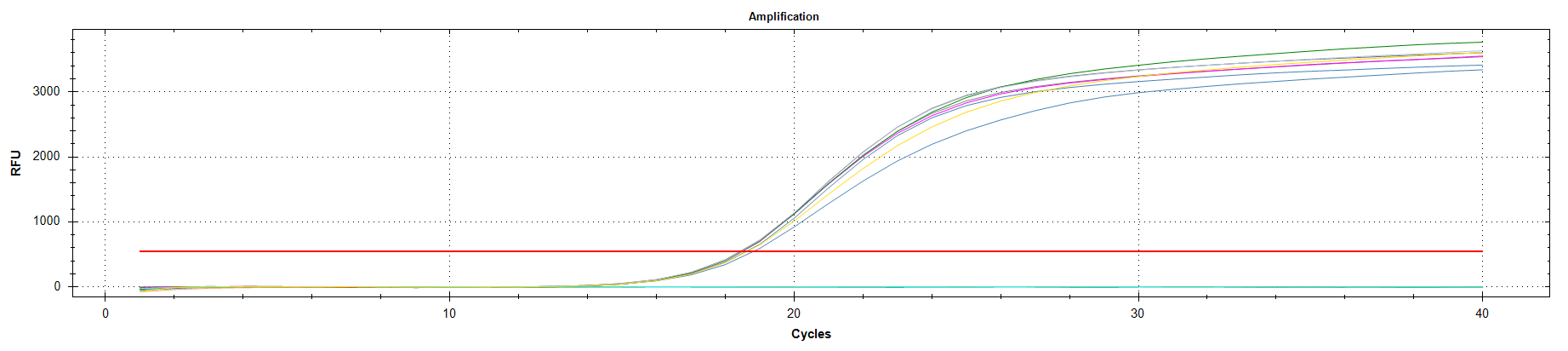


K)


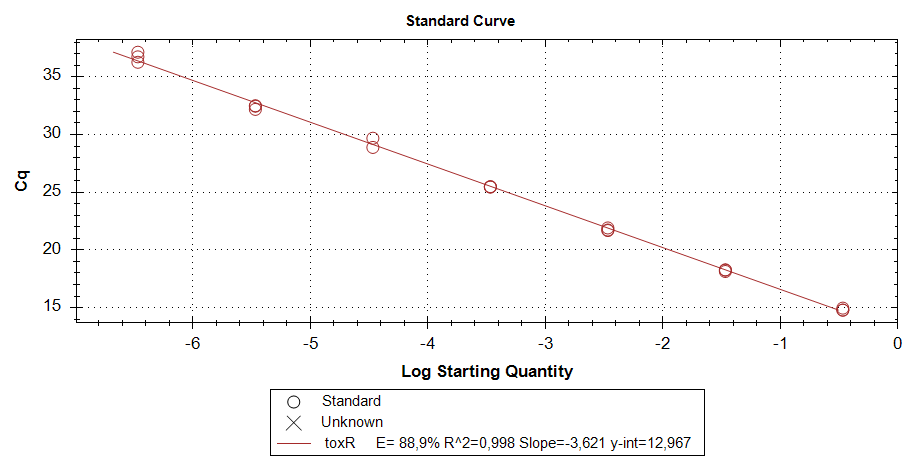


M)


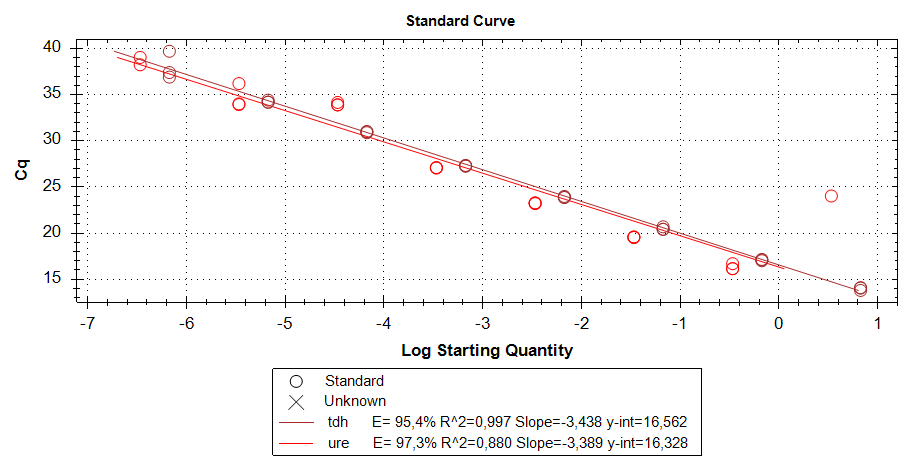


N)


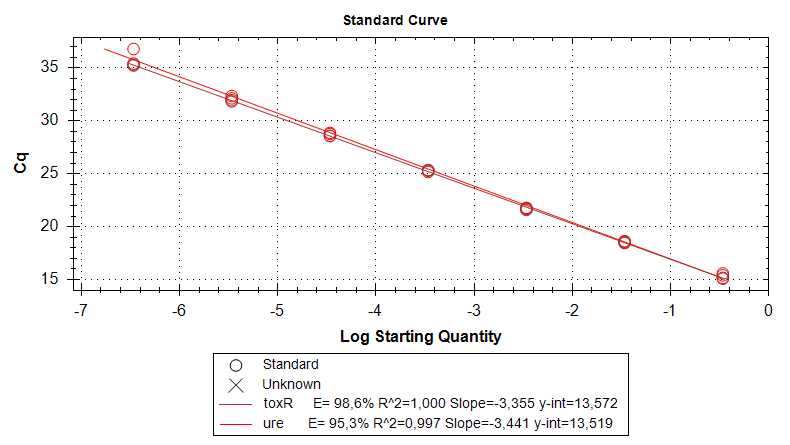


O)


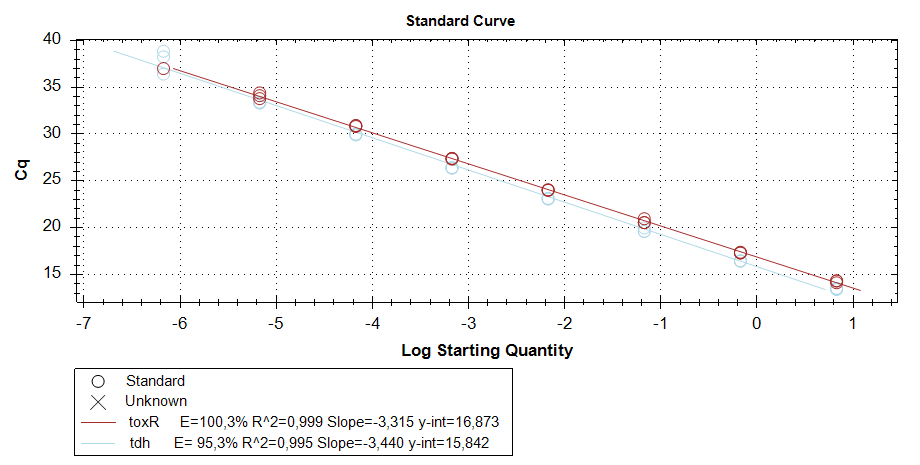


P)


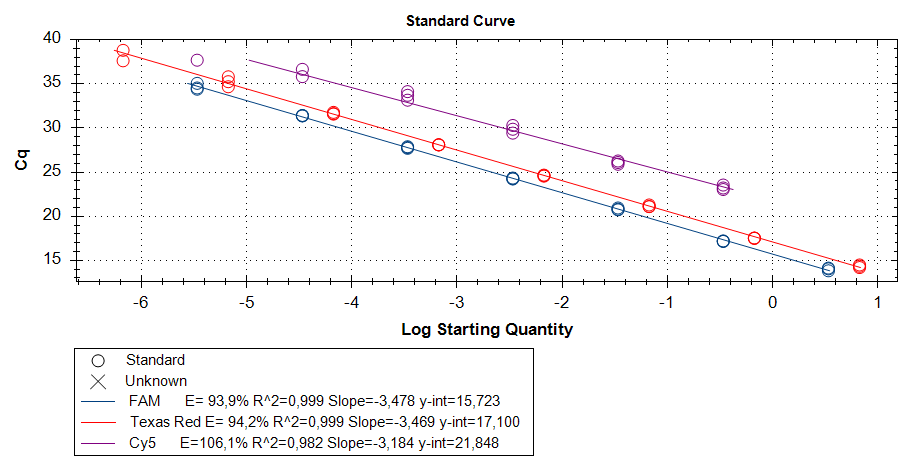


Q)

Figure S2. Experiments for qPCR optimization. Primer optimization, with melt curve analyses for *toxR* A) and B), *tdh* C) and D), and *ure* E) and F). Probe optimization for *toxR*, *tdh* and *ure* G), H) and I) respectively. Temperature optimization for *toxR*, *tdh* and *ure* J), K) and L) respectively. Amplification efficiency in simplex for *toxR* M) and *tdh* and *ure* N), and in multiplex for *toxR*-*tdh* O) and *toxR*-*ure* P) and *toxR*-*tdh-ure* Q).


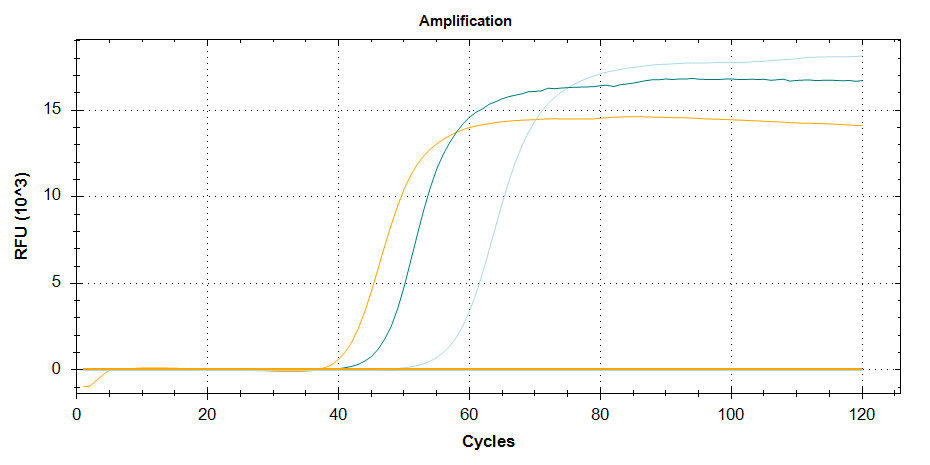


A)


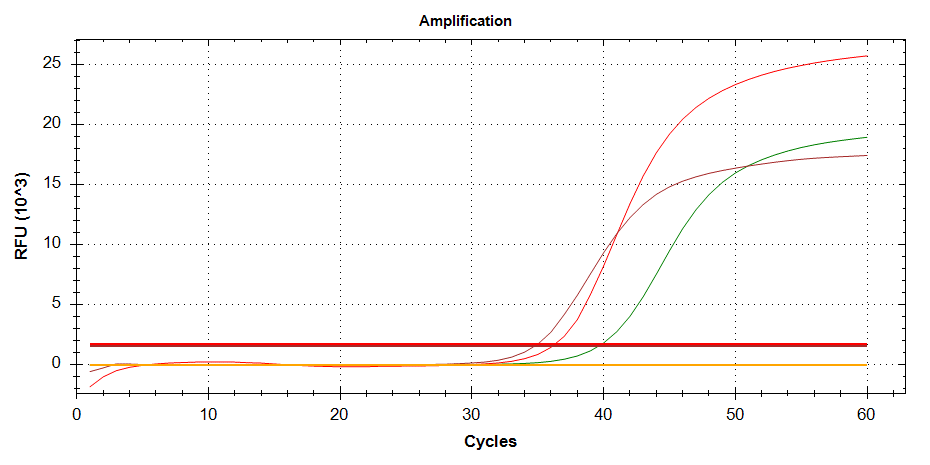


B)

D)


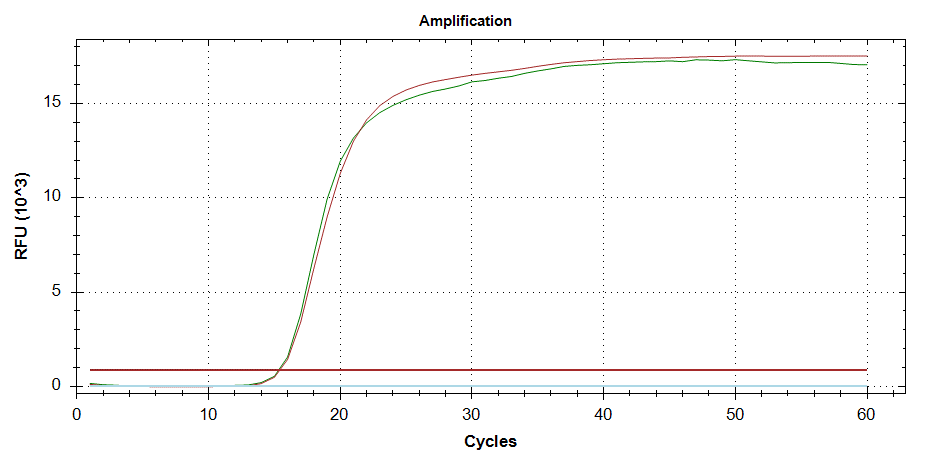

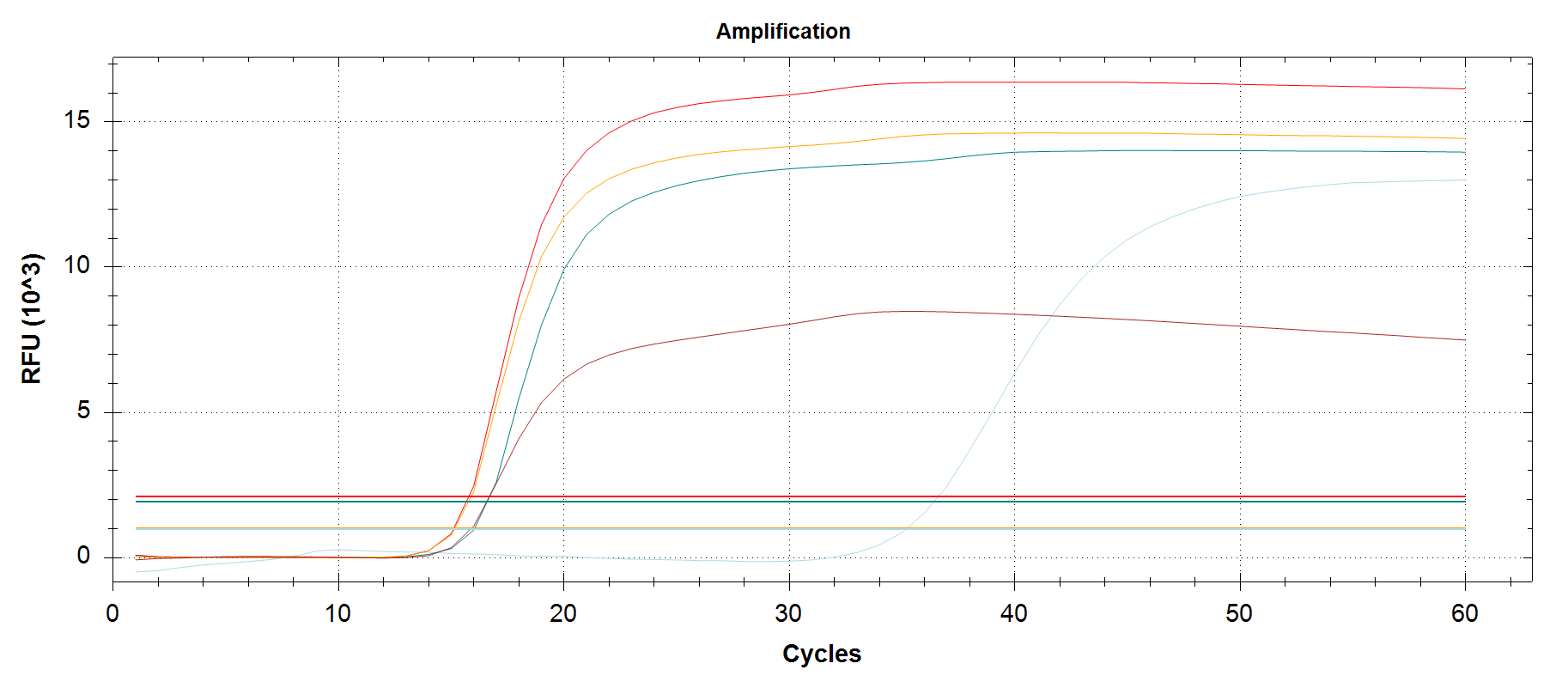


C)

Figure S3. LAMP assay optimization. Light blue, dark blue and orange indicate amplification with 600n 800 and 1000 nM FIP/BIP respectively A) Green, red and dark red indicate 1000, 1200 and 1400 nM FIP/BIP respectively B) Light blue, dark blue, orange, dark red and light red indicate 0, 200, 400, 600 and 800 nM of LF/LB respectively C). Temperature gradient from 60 to 70 °C, the red arrow indicates the amplification plot at 68 °C.

E)


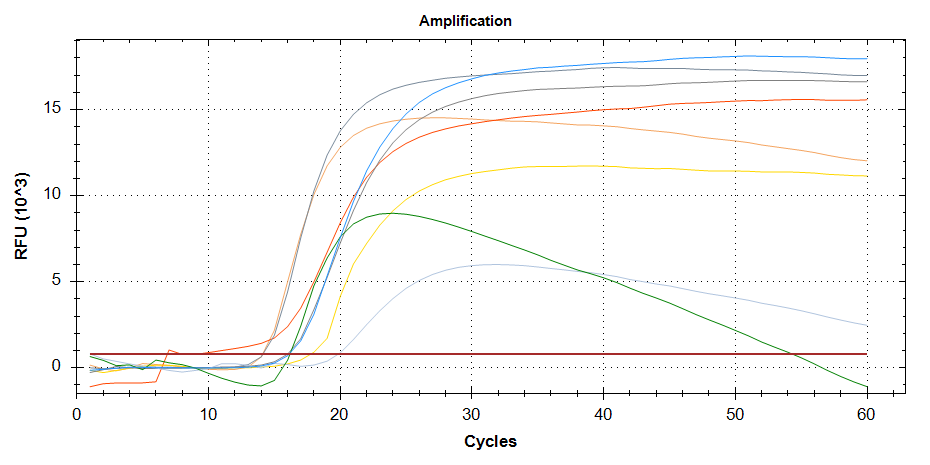


| Table S1. Number of positive partitions in LOD samples | | | | |
| --- | --- | --- | --- | --- |
| Concentration (CFU/ 25 g) | 15.4 | 7.4 | 3.7 | 1.2 |
| Number of samples | 2 | 3 | 4 | 4 |
| AbsQ* | 3/1 | 1/1/0 | 2/0/1/0 | 0/0/0/0 |
| QX | 2/0 | 0/0/0 | 3/0/0/0 | 0/0/0/0 |
| QXC | 1/3 | 0/2/1 | 0/3/2/0 | 4/0/2/1 |
| *The NTC presented 1 positive partition | | | | |
